# 3-Acetyl-1-(3-methyl­phen­yl)-5-phenyl-1*H*-pyrazole-4-carbonitrile

**DOI:** 10.1107/S160053681200181X

**Published:** 2012-01-21

**Authors:** Hatem A. Abdel-Aziz, Abdul-Rahman M. Al-Obaid, Hazem A. Ghabbour, Madhukar Hemamalini, Hoong-Kun Fun

**Affiliations:** a Department of Pharmaceutical Chemistry, College of Pharmacy, King Saud University, PO Box 2457, Riyadh 11451, Saudi Arabia; b X-ray Crystallography Unit, School of Physics, Universiti Sains Malaysia, 11800 USM, Penang, Malaysia

## Abstract

In the title compound, C_19_H_15_N_3_O, the central pyrazole ring makes dihedral angles of 35.52 (12) and 62.21 (11)° with the attached phenyl and methyl-substituted phenyl rings, respectively. The corresponding angle between the phenyl and methyl-substituted phenyl rings is 62.90 (11)°. In the crystal, mol­ecules are connected by weak C—H⋯O hydrogen bonds, forming supra­molecular chains propagating along the *a*-axis direction.

## Related literature

For details and applications of pyrazole compounds, see: Kovbasyuk *et al.* (2004 ▶); Sachse *et al.* (2008 ▶); De Geest *et al.* (2007 ▶); Roy *et al.* (2008 ▶). For related structures, see: Fun *et al.* (2011**a* ▶,*b* ▶,c* ▶). For further synthetic details, see: Nassar *et al.* (2011 ▶). 
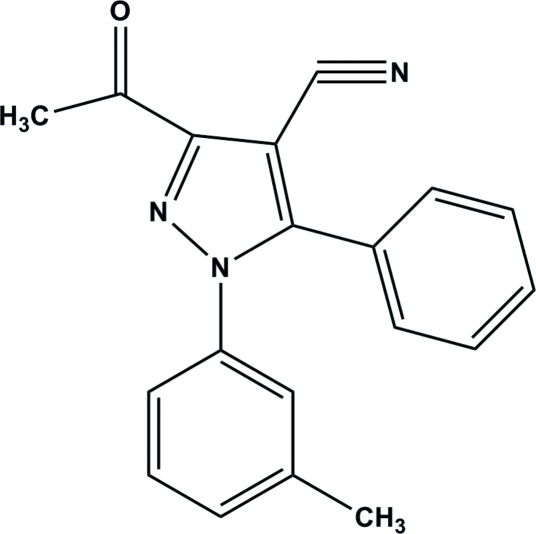


## Experimental

### 

#### Crystal data

C_19_H_15_N_3_O
*M* *_r_* = 301.34
Monoclinic, 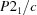

*a* = 11.8544 (8) Å
*b* = 7.6731 (6) Å
*c* = 17.4048 (15) Å
β = 96.202 (6)°
*V* = 1573.9 (2) Å^3^
*Z* = 4
Cu *K*α radiation
μ = 0.65 mm^−1^
*T* = 296 K
0.55 × 0.21 × 0.16 mm

#### Data collection

Bruker SMART APEXII CCD diffractometer
Absorption correction: multi-scan (*SADABS*; Bruker, 2009 ▶) *T*_min_ = 0.717, *T*_max_ = 0.902
8352 measured reflections
2776 independent reflections
1804 reflections with *I* > 2σ(*I*)
*R*_int_ = 0.091

#### Refinement

*R*[*F* ^2^ > 2σ(*F* ^2^)] = 0.059
*wR*(*F* ^2^) = 0.227
*S* = 1.05
2776 reflections
209 parameters
H-atom parameters constrained
Δρ_max_ = 0.27 e Å^−3^
Δρ_min_ = −0.37 e Å^−3^

### 

Data collection: *APEX2* (Bruker, 2009 ▶); cell refinement: *SAINT* (Bruker, 2009 ▶); data reduction: *SAINT*; program(s) used to solve structure: *SHELXTL* (Sheldrick, 2008 ▶); program(s) used to refine structure: *SHELXTL*; molecular graphics: *SHELXTL*; software used to prepare material for publication: *SHELXTL* and *PLATON* (Spek, 2009 ▶).

## Supplementary Material

Crystal structure: contains datablock(s) global, I. DOI: 10.1107/S160053681200181X/hb6607sup1.cif

Structure factors: contains datablock(s) I. DOI: 10.1107/S160053681200181X/hb6607Isup2.hkl

Supplementary material file. DOI: 10.1107/S160053681200181X/hb6607Isup3.cml

Additional supplementary materials:  crystallographic information; 3D view; checkCIF report

## Figures and Tables

**Table 1:** Hydrogen-bond geometry (Å, °)

*D*—H⋯*A*	*D*—H	H⋯*A*	*D*⋯*A*	*D*—H⋯*A*
C3—H3*A*⋯O1^i^	0.93	2.56	3.444 (3)	160

Symmetry code: (i) 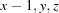.

**Fig. 1.**
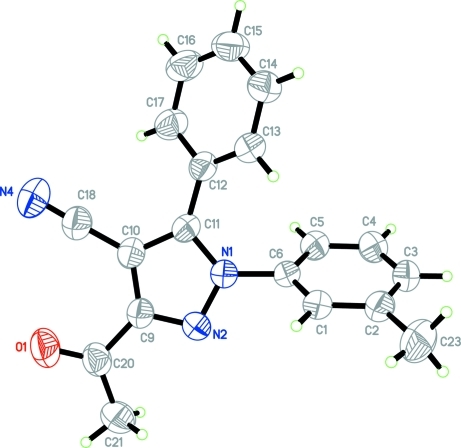
The asymmetric unit of the title compound, showing 50% probability displacement ellipsoids.

**Fig. 2.**
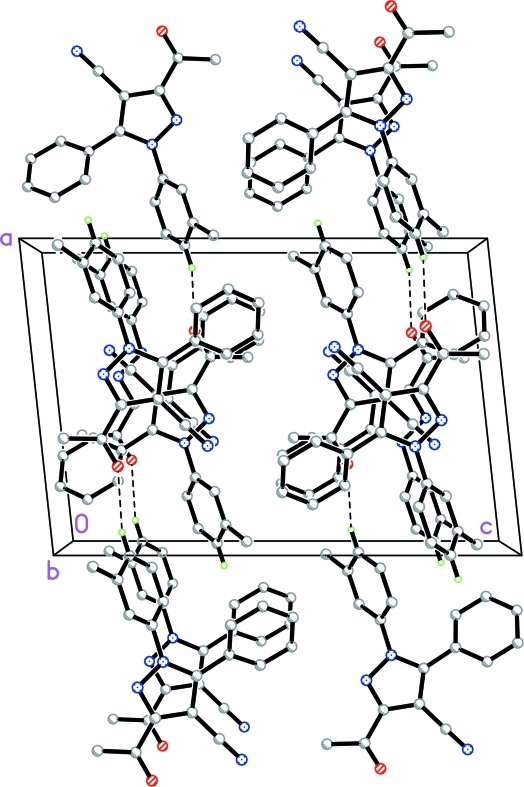
The crystal packing of title compound (I).

## supplementary crystallographic information

### Comment

Pyrazole-based ligands have attracted considerable attention due to their bridging nature and possibility for easy functionalization with various additional donor groups (Kovbasyuk *et al.*, 2004; Sachse *et al.*, 2008). In particular, azomethine-functionalized pyrazoles have been used extensively as ligands in the field of coordination chemistry and catalysis (De Geest *et al.*, 2007; Roy *et al.*, 2008). The crystal structures of 4-(1,3-Diphenyl-4,5-dihydro-1*H*-pyrazol-5-yl)-1,3-diphenyl- 1*H*-pyrazole, 3-Methyl-5-oxo-4-(2-phenylhydrazinylidene)-4,5- dihydro-1*H*-pyrazole-1-carbothioamide and (2*E*)-3-(1,3- Diphenyl-1*H*-pyrazol-4-yl)-1-phenylprop-2-en-1-one (Fun *et al.*, 2011**a*,*b*,c*) have been reported from our laboratory. In continuation of our studies of pyrazole compounds, the crystal structure determination of the title compound has been undertaken.

The asymmetric unit of the title compound is shown in Fig. 1. The central pyrazole (N1,N2/C9–C11) ring makes dihedral angles of 35.52 (12) and 62.21 (11)° with the attached phenyl (C12–C17) and methyl substituted pheny (C1–C6) rings. The corresponding angle between the phenyl (C12–C17) and methyl substituted (C1–C6) phenyl rings is 62.90 (11)°.

In the crystal structure (Fig. 2), molecules are connected by weak intermolecular C—H···O (Table 1) hydrogen bonds, forming supramolecular chains propagating along the *a*-axis direction.

### Experimental

The title compound was prepared according to the reported method (Nassar *et al.*, 2011). Colourless blocks were obtained by slowly evaporating from ethanol at room temperature.

### Refinement

All hydrogen atoms were positioned geometrically [C–H = 0.93 or 0.96 Å] and were refined using a riding model, with *U*_iso_(H) = 1.2 or 1.5 *U*_eq_(C). A rotating group model was applied to the methyl groups.

### Crystal data

**Table:**

C_19_H_15_N_3_O	*F*(000) = 632
*M**_r_* = 301.34	*D*_x_ = 1.272 Mg m^−^^3^
Monoclinic, *P*2_1_/*c*	Cu *K*α radiation, λ = 1.54178 Å
Hall symbol: -P 2ybc	Cell parameters from 1651 reflections
*a* = 11.8544 (8) Å	θ = 6.3–66.5°
*b* = 7.6731 (6) Å	µ = 0.65 mm^−^^1^
*c* = 17.4048 (15) Å	*T* = 296 K
β = 96.202 (6)°	Block, colourless
*V* = 1573.9 (2) Å^3^	0.55 × 0.21 × 0.16 mm
*Z* = 4	

### Data collection

**Table:**

Bruker SMART APEXII CCD diffractometer	2776 independent reflections
Radiation source: fine-focus sealed tube	1804 reflections with *I* > 2σ(*I*)
graphite	*R*_int_ = 0.091
φ and ω scans	θ_max_ = 67.4°, θ_min_ = 3.8°
Absorption correction: multi-scan (*SADABS*; Bruker, 2009)	*h* = −11→14
*T*_min_ = 0.717, *T*_max_ = 0.902	*k* = −9→8
8352 measured reflections	*l* = −19→20

### Refinement

**Table:**

Refinement on *F*^2^	Secondary atom site location: difference Fourier map
Least-squares matrix: full	Hydrogen site location: inferred from neighbouring sites
*R*[*F*^2^ > 2σ(*F*^2^)] = 0.059	H-atom parameters constrained
*wR*(*F*^2^) = 0.227	*w* = 1/[σ^2^(*F*_o_^2^) + (0.1264*P*)^2^ + 0.0573*P*] where *P* = (*F*_o_^2^ + 2*F*_c_^2^)/3
*S* = 1.05	(Δ/σ)_max_ < 0.001
2776 reflections	Δρ_max_ = 0.27 e Å^−^^3^
209 parameters	Δρ_min_ = −0.37 e Å^−^^3^
0 restraints	Extinction correction: *SHELXTL* (Sheldrick, 2008), Fc^*^=kFc[1+0.001xFc^2^λ^3^/sin(2θ)]^-1/4^
Primary atom site location: structure-invariant direct methods	Extinction coefficient: 0.0042 (13)

### Special details

**Table:**

Geometry. All s.u.'s (except the s.u. in the dihedral angle between two l.s. planes) are estimated using the full covariance matrix. The cell s.u.'s are taken into account individually in the estimation of s.u.'s in distances, angles and torsion angles; correlations between s.u.'s in cell parameters are only used when they are defined by crystal symmetry. An approximate (isotropic) treatment of cell s.u.'s is used for estimating s.u.'s involving l.s. planes.
Refinement. Refinement of F^2^ against ALL reflections. The weighted R-factor wR and goodness of fit S are based on F^2^, conventional R-factors R are based on F, with F set to zero for negative F^2^. The threshold expression of F^2^ > 2σ(F^2^) is used only for calculating R-factors(gt) etc. and is not relevant to the choice of reflections for refinement. R-factors based on F^2^ are statistically about twice as large as those based on F, and R- factors based on ALL data will be even larger.

### Fractional atomic coordinates and isotropic or equivalent isotropic displacement parameters (Å^2^)

**Table:**

	*x*	*y*	*z*	*U*_iso_*/*U*_eq_	
C1	0.18649 (19)	−0.0026 (3)	0.85181 (12)	0.0503 (7)	
H1A	0.2331	−0.0955	0.8688	0.060*	
C2	0.0758 (2)	0.0011 (3)	0.86879 (13)	0.0550 (7)	
C3	0.0088 (2)	0.1432 (3)	0.84253 (14)	0.0586 (7)	
H3A	−0.0662	0.1492	0.8534	0.070*	
C4	0.0527 (2)	0.2746 (3)	0.80077 (14)	0.0590 (7)	
H4A	0.0064	0.3677	0.7837	0.071*	
C5	0.1629 (2)	0.2711 (3)	0.78384 (13)	0.0538 (6)	
H5A	0.1924	0.3604	0.7559	0.065*	
C6	0.22883 (19)	0.1295 (3)	0.80997 (12)	0.0461 (6)	
N1	0.34545 (16)	0.1197 (2)	0.79402 (11)	0.0489 (6)	
N2	0.42757 (17)	0.1217 (2)	0.85538 (11)	0.0523 (6)	
C9	0.5249 (2)	0.1171 (3)	0.82443 (14)	0.0482 (6)	
C10	0.5062 (2)	0.1118 (3)	0.74350 (13)	0.0496 (7)	
C11	0.38934 (19)	0.1128 (2)	0.72492 (13)	0.0458 (6)	
C12	0.32137 (19)	0.1023 (3)	0.64932 (13)	0.0480 (6)	
C13	0.2211 (2)	0.0089 (3)	0.63772 (13)	0.0521 (6)	
H13A	0.1937	−0.0474	0.6792	0.062*	
C14	0.1610 (2)	−0.0016 (3)	0.56516 (14)	0.0615 (7)	
H14A	0.0931	−0.0634	0.5583	0.074*	
C15	0.2016 (2)	0.0791 (4)	0.50341 (16)	0.0684 (8)	
H15A	0.1609	0.0724	0.4547	0.082*	
C16	0.3012 (2)	0.1689 (3)	0.51313 (15)	0.0687 (8)	
H16A	0.3289	0.2213	0.4708	0.082*	
C17	0.3613 (2)	0.1829 (3)	0.58552 (13)	0.0588 (7)	
H17A	0.4286	0.2462	0.5918	0.071*	
C18	0.5895 (2)	0.1047 (3)	0.69062 (17)	0.0593 (7)	
N4	0.6559 (2)	0.1033 (3)	0.64697 (16)	0.0835 (8)	
C20	0.6343 (2)	0.1168 (3)	0.87444 (16)	0.0569 (7)	
O1	0.72089 (16)	0.0876 (3)	0.84499 (12)	0.0754 (6)	
C23	0.0279 (3)	−0.1453 (4)	0.91259 (19)	0.0857 (10)	
H23A	0.0861	−0.2303	0.9263	0.129*	
H23D	−0.0337	−0.1989	0.8808	0.129*	
H23B	0.0008	−0.1002	0.9587	0.129*	
C21	0.6332 (2)	0.1535 (4)	0.95825 (15)	0.0720 (8)	
H21A	0.7093	0.1493	0.9834	0.108*	
H21B	0.5877	0.0677	0.9806	0.108*	
H21C	0.6018	0.2672	0.9648	0.108*	

### Atomic displacement parameters (Å^2^)

**Table:**

	*U*^11^	*U*^22^	*U*^33^	*U*^12^	*U*^13^	*U*^23^
C1	0.0445 (16)	0.0626 (13)	0.0437 (14)	0.0026 (10)	0.0046 (11)	0.0018 (9)
C2	0.0493 (16)	0.0690 (14)	0.0472 (13)	−0.0093 (11)	0.0078 (11)	−0.0001 (10)
C3	0.0421 (15)	0.0816 (16)	0.0528 (15)	0.0036 (11)	0.0086 (12)	−0.0018 (11)
C4	0.0484 (16)	0.0692 (14)	0.0592 (16)	0.0083 (12)	0.0045 (12)	0.0040 (11)
C5	0.0506 (15)	0.0614 (13)	0.0499 (14)	0.0022 (11)	0.0078 (11)	0.0052 (10)
C6	0.0397 (15)	0.0631 (12)	0.0358 (13)	0.0000 (9)	0.0048 (10)	−0.0027 (9)
N1	0.0401 (12)	0.0642 (11)	0.0426 (12)	0.0001 (8)	0.0054 (9)	−0.0009 (8)
N2	0.0423 (13)	0.0694 (12)	0.0443 (12)	−0.0013 (9)	0.0009 (10)	−0.0006 (8)
C9	0.0412 (15)	0.0551 (12)	0.0491 (15)	−0.0021 (9)	0.0083 (11)	−0.0004 (9)
C10	0.0449 (15)	0.0538 (12)	0.0515 (15)	0.0006 (9)	0.0112 (11)	−0.0009 (9)
C11	0.0479 (16)	0.0495 (11)	0.0413 (14)	−0.0013 (9)	0.0105 (11)	0.0015 (8)
C12	0.0470 (15)	0.0528 (12)	0.0452 (14)	0.0058 (9)	0.0100 (11)	−0.0015 (8)
C13	0.0524 (15)	0.0598 (13)	0.0446 (13)	0.0006 (11)	0.0080 (11)	−0.0033 (9)
C14	0.0573 (17)	0.0707 (15)	0.0553 (15)	0.0001 (12)	0.0007 (13)	−0.0077 (12)
C15	0.074 (2)	0.0811 (17)	0.0479 (16)	0.0099 (14)	−0.0039 (14)	−0.0015 (12)
C16	0.083 (2)	0.0761 (16)	0.0487 (16)	0.0035 (14)	0.0133 (14)	0.0132 (12)
C17	0.0654 (17)	0.0610 (14)	0.0510 (15)	−0.0043 (11)	0.0112 (12)	0.0052 (10)
C18	0.0510 (18)	0.0670 (15)	0.0610 (18)	−0.0022 (11)	0.0110 (14)	−0.0011 (11)
N4	0.0713 (18)	0.1056 (18)	0.0788 (19)	0.0031 (13)	0.0314 (15)	0.0018 (13)
C20	0.0425 (16)	0.0623 (14)	0.0655 (17)	−0.0066 (11)	0.0044 (13)	0.0035 (11)
O1	0.0429 (13)	0.0973 (14)	0.0857 (15)	−0.0028 (9)	0.0053 (11)	−0.0070 (10)
C23	0.077 (2)	0.096 (2)	0.088 (2)	−0.0160 (16)	0.0272 (18)	0.0198 (16)
C21	0.0598 (17)	0.0930 (19)	0.0603 (17)	−0.0084 (14)	−0.0071 (14)	−0.0044 (14)

### Geometric parameters (Å, °)

**Table:**

C1—C6	1.375 (3)	C12—C13	1.384 (3)
C1—C2	1.376 (3)	C12—C17	1.398 (3)
C1—H1A	0.9300	C13—C14	1.384 (3)
C2—C3	1.396 (3)	C13—H13A	0.9300
C2—C23	1.503 (4)	C14—C15	1.372 (4)
C3—C4	1.377 (3)	C14—H14A	0.9300
C3—H3A	0.9300	C15—C16	1.362 (4)
C4—C5	1.370 (3)	C15—H15A	0.9300
C4—H4A	0.9300	C16—C17	1.383 (4)
C5—C6	1.386 (3)	C16—H16A	0.9300
C5—H5A	0.9300	C17—H17A	0.9300
C6—N1	1.441 (3)	C18—N4	1.151 (3)
N1—C11	1.362 (3)	C20—O1	1.217 (3)
N1—N2	1.365 (3)	C20—C21	1.487 (4)
N2—C9	1.325 (3)	C23—H23A	0.9600
C9—C10	1.403 (3)	C23—H23D	0.9600
C9—C20	1.482 (3)	C23—H23B	0.9600
C10—C11	1.388 (3)	C21—H21A	0.9600
C10—C18	1.422 (4)	C21—H21B	0.9600
C11—C12	1.469 (3)	C21—H21C	0.9600
			
C6—C1—C2	120.6 (2)	C17—C12—C11	119.2 (2)
C6—C1—H1A	119.7	C14—C13—C12	120.8 (2)
C2—C1—H1A	119.7	C14—C13—H13A	119.6
C1—C2—C3	117.9 (2)	C12—C13—H13A	119.6
C1—C2—C23	121.0 (2)	C15—C14—C13	120.0 (2)
C3—C2—C23	121.2 (2)	C15—C14—H14A	120.0
C4—C3—C2	120.7 (2)	C13—C14—H14A	120.0
C4—C3—H3A	119.6	C16—C15—C14	120.3 (2)
C2—C3—H3A	119.6	C16—C15—H15A	119.9
C5—C4—C3	121.5 (2)	C14—C15—H15A	119.9
C5—C4—H4A	119.3	C15—C16—C17	120.5 (2)
C3—C4—H4A	119.3	C15—C16—H16A	119.8
C4—C5—C6	117.5 (2)	C17—C16—H16A	119.8
C4—C5—H5A	121.3	C16—C17—C12	120.2 (2)
C6—C5—H5A	121.3	C16—C17—H17A	119.9
C1—C6—C5	121.8 (2)	C12—C17—H17A	119.9
C1—C6—N1	118.43 (19)	N4—C18—C10	178.1 (3)
C5—C6—N1	119.8 (2)	O1—C20—C9	118.5 (2)
C11—N1—N2	112.52 (19)	O1—C20—C21	123.0 (2)
C11—N1—C6	129.64 (18)	C9—C20—C21	118.4 (3)
N2—N1—C6	117.81 (19)	C2—C23—H23A	109.5
C9—N2—N1	105.08 (19)	C2—C23—H23D	109.5
N2—C9—C10	111.1 (2)	H23A—C23—H23D	109.5
N2—C9—C20	120.4 (2)	C2—C23—H23B	109.5
C10—C9—C20	128.5 (3)	H23A—C23—H23B	109.5
C11—C10—C9	106.2 (2)	H23D—C23—H23B	109.5
C11—C10—C18	126.5 (2)	C20—C21—H21A	109.5
C9—C10—C18	127.3 (2)	C20—C21—H21B	109.5
N1—C11—C10	105.15 (19)	H21A—C21—H21B	109.5
N1—C11—C12	124.6 (2)	C20—C21—H21C	109.5
C10—C11—C12	130.2 (2)	H21A—C21—H21C	109.5
C13—C12—C17	118.2 (2)	H21B—C21—H21C	109.5
C13—C12—C11	122.6 (2)		
			
C6—C1—C2—C3	−0.3 (3)	C6—N1—C11—C10	177.65 (19)
C6—C1—C2—C23	178.3 (2)	N2—N1—C11—C12	177.50 (17)
C1—C2—C3—C4	0.2 (3)	C6—N1—C11—C12	−4.4 (3)
C23—C2—C3—C4	−178.4 (3)	C9—C10—C11—N1	0.4 (2)
C2—C3—C4—C5	−0.3 (4)	C18—C10—C11—N1	179.7 (2)
C3—C4—C5—C6	0.4 (4)	C9—C10—C11—C12	−177.45 (19)
C2—C1—C6—C5	0.4 (3)	C18—C10—C11—C12	1.9 (3)
C2—C1—C6—N1	179.76 (18)	N1—C11—C12—C13	−36.0 (3)
C4—C5—C6—C1	−0.5 (3)	C10—C11—C12—C13	141.5 (2)
C4—C5—C6—N1	−179.78 (19)	N1—C11—C12—C17	146.9 (2)
C1—C6—N1—C11	119.3 (2)	C10—C11—C12—C17	−35.6 (3)
C5—C6—N1—C11	−61.3 (3)	C17—C12—C13—C14	−1.1 (3)
C1—C6—N1—N2	−62.6 (2)	C11—C12—C13—C14	−178.3 (2)
C5—C6—N1—N2	116.7 (2)	C12—C13—C14—C15	0.9 (4)
C11—N1—N2—C9	0.4 (2)	C13—C14—C15—C16	0.3 (4)
C6—N1—N2—C9	−177.99 (17)	C14—C15—C16—C17	−1.3 (4)
N1—N2—C9—C10	−0.1 (2)	C15—C16—C17—C12	1.1 (4)
N1—N2—C9—C20	−179.71 (19)	C13—C12—C17—C16	0.2 (3)
N2—C9—C10—C11	−0.2 (2)	C11—C12—C17—C16	177.4 (2)
C20—C9—C10—C11	179.4 (2)	N2—C9—C20—O1	169.6 (2)
N2—C9—C10—C18	−179.5 (2)	C10—C9—C20—O1	−9.9 (3)
C20—C9—C10—C18	0.0 (4)	N2—C9—C20—C21	−10.8 (3)
N2—N1—C11—C10	−0.5 (2)	C10—C9—C20—C21	169.7 (2)

### Hydrogen-bond geometry (Å, °)

**Table:**

*D*—H···*A*	*D*—H	H···*A*	*D*···*A*	*D*—H···*A*
C3—H3A···O1^i^	0.93	2.56	3.444 (3)	160

Symmetry codes: (i) *x*−1, *y*, *z*.
